# TGFβ1 – induced recruitment of human bone mesenchymal stem cells is mediated by the primary cilium in a SMAD3-dependent manner

**DOI:** 10.1038/srep35542

**Published:** 2016-10-17

**Authors:** Marie-Noëlle Labour, Mathieu Riffault, Søren T. Christensen, David A. Hoey

**Affiliations:** 1 Trinity Centre for Bioengineering, Trinity Biomedical Sciences Institute, Trinity College Dublin, Dublin, Ireland; 2 Department of Mechanical and Manufacturing Engineering, School of Engineering, Trinity College Dublin, Dublin, Ireland; 3 Department of Mechanical, Aeronautical and Biomedical Engineering, University of Limerick, Limerick, Ireland; 4 Advanced Materials and Bioengineering Research Centre, Trinity College Dublin & RCSI, Dublin 2, Ireland; 5 Department of Biology, University of Copenhagen, Copenhagen, Denmark

## Abstract

The recruitment of mesenchymal stem cells (MSCs) is a crucial process in the development, maintenance and repair of tissues throughout the body. Transforming growth factor-β1 (TGFβ1) is a potent chemokine essential for the recruitment of MSCs in bone, coupling the remodelling cycle. The primary cilium is a sensory organelle with important roles in bone and has been associated with cell migration and more recently TGFβ signalling. Dysregulation of TGFβ signalling or cilia has been linked to a number of skeletal pathologies. Therefore, this study aimed to determine the role of the primary cilium in TGFβ1 signalling and associated migration in human MSCs. In this study we demonstrate that low levels of TGFβ1 induce the recruitment of MSCs, which relies on proper formation of the cilium. Furthermore, we demonstrate that receptors and downstream signalling components in canonical TGFβ signalling localize to the cilium and that TGFβ1 signalling is associated with activation of SMAD3 at the ciliary base. These findings demonstrate a novel role for the primary cilium in the regulation of TGFβ signalling and subsequent migration of MSCs, and highlight the cilium as a target to manipulate this key pathway and enhance MSC recruitment for the treatment of skeletal diseases.

The adult skeleton is a dynamic structure that is continuously adapting and remodelling to meet the demands of the local biochemical and biophysical environment. This is achieved through tightly regulated and coordinated bone resorption by osteoclasts followed by bone formation by mesenchymal derived osteoblasts. Due to the non-proliferative state and short lifespan of the osteoblast, continued bone formation requires the replenishment of the exhausted osteoblast from a mesenchymal stem cell (MSC) population^1^. Tight coordination of this process is essential as defects in MSC recruitment have been associated with a number of skeletal pathologies including osteoporosis. Indeed, osteoporotic patients have reduced defect and fracture healing rates^2^,^3^,^4^ and transplanted osteoporotic MSCs demonstrate significantly reduced homing to a fracture site^4^. Deciphering the mechanisms by which MSCs are recruited to sites of bone formation is critical to the development of novel anabolic therapies for osteoporosis and to tissue engineering strategies for treating bone defects and fractures.

Primary cilia are membrane-bound, microtubule-based organelles that extend as solitary structures from a modified centriole (basal body) at the surface of most mammalian cell types^5^, including human MSCs (hMSCs)^6^,^7^. Primary cilia function as unique sensory units with a diverse complement of receptors and ion channels that detect extracellular cues and transmit signalling information to the cell to control cellular and physiological processes during development and in tissue homeostasis^7^,^8^,^9^,^10^. Primary cilia are assembled and maintained by a process known as intraflagellar transport (IFT)^11^, which when defective, leads to aberrant cell signalling and numerous clinical disorders termed ciliopathies, including skeletal abnormalities^12^,^13^. Indeed, several studies have demonstrated an important role for the primary cilium in adult bone where the organelle is required for loading-induced osteogenesis^14^,^15^,^16^,^17^,^18^,^19^,^20^. In mesenchymal progenitors the cilium is also involved in biochemical and biophysical sensing that dictates early osteogenic responses^6^,^21^,^22^,^23^ and acts as a signaling hub regulating Wnt signaling, a critical pathway in skeletal tissue^24^. Recently, Chen *et al.* demonstrated that bone formation, in response to loading, was significantly inhibited in mice subjected to a conditional knock-out of the primary cilium within the bone marrow, including the stem cell population^25^. Interestingly, this occurred due to a decrease in the area of active mineralizing surface rather than the rate of mineralization suggesting that the cilium may be involved in osteoprogenitor cell recruitment. Similarly, the primary cilium plays a critical role in regulating directional cell migration in fibroblasts and endothelial cells, with mice with defective cilia exhibiting significantly reduced wound healing rates due to a decrease in cell recruitment to sites of injury^26^,^27^.

Transforming Growth Factor Beta 1 (TGFβ1) is a ubiquitous growth factor in skeletal tissue, playing major roles in development and maintenance of bone metabolism through the control of cellular proliferation, differentiation, matrix deposition and migration^28^. In bone, TGFβ1 is stored in the extracellular matrix in a latent form and is activated during osteoclast-mediated resorption^29^, and is released in response to loading^30^, and as a result of fracture^31^,^32^. Tang *et al.*^29^ demonstrated that TGFβ1 is essential for the recruitment of MSCs to the bone surface, whereby TGFβ1 is released by osteoclast mediated resorption generating a gradient that induces the recruitment of MSCs in a SMAD3-dependent manner. A number of skeletal pathologies such as osteopenia, osteoarthritis or Camurati-Engelmann disease have been associated with a deficiency or overexpression of TGFβ1 and associated signalling^29^,^33^,^34^,^35^,^36^. These studies highlight the critical role of TGFβ1 in maintaining bone homeostasis, but also importantly, the need for tight regulation of this pathway. TGFβ1 signals through a heteromeric complex, within which the TGFβ type II receptor (TGFβRII) transphosphorylates and activates the type I receptor (TGFβRI). TGFβRI in turn initiates SMAD signalling by phosphorylating SMAD2/3, followed by translocation of same to the nucleus^37^. TGFβ receptors have been shown to undergo regulated microdomain clustering, multimerisation and internalisation, mechanisms that affect TGFβ1 binding and downstream effector recruitment and therefore play crucial roles in the regulation of signal intensity and duration^38^,^39^. Very recent studies have elegantly demonstrated discrete spatial organisation of the TGFβ receptors within focal adhesions^40^ and intriguingly in and around the primary cilium in fibroblasts and human embryonic stem cells (hESC) as well as in a P19.CL6 mouse cancer stem cell line^41^,^42^. In fibroblasts, stimulation with TGFβ1 leads to accumulation and clathrin-mediated internalization of the receptors at the ciliary pocket for activation of SMAD2/3, which is increased in stem cells undergoing cardiomyogenesis^41^.

As MSC recruitment is pivotal for bone maintenance and repair and may be targeted as potential therapy to treat skeletal defects and disease, this study aimed to determine the role of the primary cilium in human MSC recruitment and elucidate the mechanisms by which the cilium may mediate this process. In this study, we demonstrate that TGFβ1 significantly induces human MSC recruitment in a concentration-dependent manner and that TGFβ1-induced recruitment is regulated by the primary cilium. Furthermore, we show that the cilium mediates this response through distinct spatial localisation of TGFβ receptors and signalling components at the primary cilium, whereby TGFβ1-mediated activation of SMAD3 takes place at the ciliary base. This study therefore demonstrates that signalling through the primary cilium is necessary for the sensing of a TGFβ1 chemotactic gradient in human bone mesenchymal stem cells demonstrating a novel mechanism of TGFβ signal regulation and recruitment in stem cells, and highlights the primary cilium as a potential target to enhance MSC recruitment and bone formation in orthopaedic disease.

## Results

### TGFβ1 induces recruitment and inhibits osteogenesis of human MSCs at low concentrations

**Figure 1:**
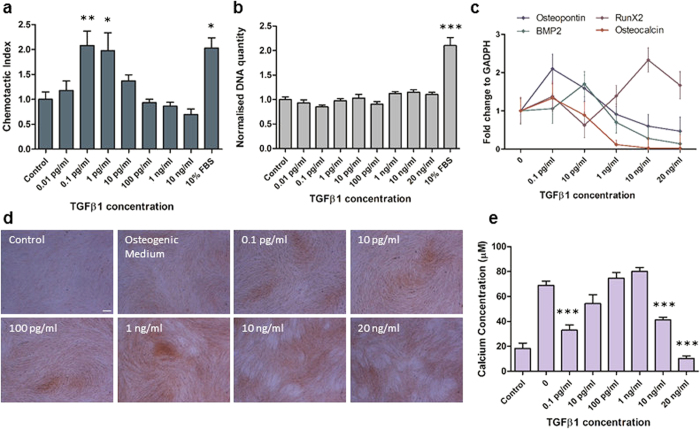
Effect of TGFβ1 on hMSC migration, proliferation and differentiation. (**a**) Chemoatxis of hMSC in response to 0.01 pg/ml to 10 ng/ml TGFβ1 expressed as chemotactic index (number of cells per field of view normalised to the control). (**b**) Proliferation of hMSC treated with 0.1 pg/ml −20 ng/ml TGFβ1 expressed as DNA concentration. (**c–e**) Osteogenic differentiation of hMSCs after 14 days of treatment with 0.1 pg/ml −20 ng/ml TGFβ1. (**c**) qPCR analysis of Osteocalcin, Osteopontin, RunX2 and BMP2 expression. (**d**) Alizarin red staining, scale bar 200 μm. (**e**) Assessment of mineralisation by calcium concentration measurement. All results are expressed as mean ± SEM and analysed using 1 way Anova with Dunnett’s multiple comparison post-test, *p < 0.05, **p < 0.01, ***p < 0.001.

As there are a limited number of often conflicting studies on the effect of TGFβ1 on hMSC behaviour^28^,^43^, we first examined the effect of TGFβ1 concentration on human MSC migration, proliferation and osteogenic differentiation (Fig. 1). With regards to hMSC recruitment, the effect of TGFβ1 concentration revealed a bell shaped profile centred on low concentrations promoting a 2.1 and 2-fold increase in hMSC migration for 0.1 and 1 pg/ml TGFβ1 respectively when compared to unstimulated control (p < 0.01 and p < 0.05, *N* = 4) (Fig. 1a). The degree of migration at these concentrations was equivalent to 10% serum demonstrating the potent chemotactic properties of TGFβ1. With regards to hMSC proliferation, TGFβ1 did not induce proliferation at any concentration investigated after 3 days (Fig. 1b). The control with 10% FBS demonstrated a significant 2.1-fold increase in proliferation (p < 0.001, *N* = 9), indicating that hMSCs can proliferate during this time interval and suggests that TGFβ1 (0.01–20,000 pg/ml) does not mediate hMSC proliferation. With regards to hMSC osteogenic differentiation, TGFβ1 was found to have a negative effect on osteogenesis at low and high concentrations. Quantitative real-time PCR analysis revealed no significant changes in RunX2, Osteopontin, BMP2 and Osteocalcin gene expression with increasing TGFβ1 concentration after 14 days treatment (Fig. 1c). Despite a lack of significance, Osteopontin expression is slightly increased at 0.1 pg/ml TGFβ1 compared to control cells, while, with the exception of RunX2, the expression of all other genes analysed were decreased with TGFβ1 concentrations greater than 1 ng/ml, suggesting an inhibition of osteogenesis with TGFβ1 treatment. To confirm these results and to assess mineral deposition, Alizarin Red staining and calcium quantification was performed after 14 days treatment. Regarding calcium staining, there is no obvious visual difference with increasing TGFβ1 treatment (Fig. 1d). However, the quantities of calcium measured after extraction were significantly decreased for 0.1 pg/ml, 10 and 20 ng/ml (Fig. 1e) when compared to unstimulated controls, confirming that low and high TGFβ1 concentrations inhibit osteogenic differentiation of hMSCs. Altogether, these data demonstrate that TGFβ1 induces recruitment, does not influence proliferation, and inhibits osteogenesis of hMSCs in a concentration-dependent manner.

### TGFβ receptors are localised and concentrated within the sub-compartment of the primary cilium

**Figure 2:**
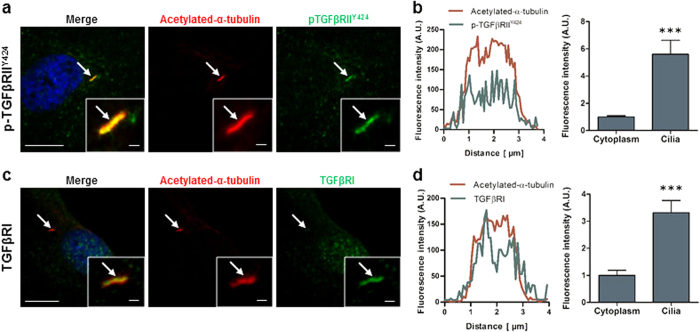
TGFβ receptors localise to the primary cilia in human MSC. (**a,b**) Immunocytochemistry analysis of the cilia (identified by arrows) using anti-acetylated-α-tubulin in red, constitutively active TGFβRII (p-TGFβRII^Y424^) (**a**) and TGFβRI (**b**) in green and nucleus in blue (DAPI). Scale bar 5 μm and 1 μm in the enlarged pictures. (**c,d**) Fluorescence intensity analysis of the levels of p-TGFβRII^Y424^ (**c**) and TGFβRI (**d**). Intensity profile of Acetylated-α-tubulin (red) and receptors (green) along the cilia (left panel) and analysis of the average intensity in the cilia and cytoplasm (right panel). Statistical analysis using Student’s *t*-test, ***p < 0.001.

Given the potent role of TGFβ1 in mediating hMSC migration demonstrated above, and the potential role of the primary cilium in regulating MSC recruitment *in vivo*^25^, we next examined whether the primary cilium may act as a chemosensor for the TGFβ1 ligand. To this end, we performed immunofluorescence microscopy (IFM) analysis on the localization of TGFβRI as well as TGFβRII constitutively autophosphorylated on tyrosine residue 424 (p-TGFβRII^Y424^), which is required for receptor activity^44^. Following 48 hours of serum starvation to promote ciliogenesis, IFM was performed with antibodies against TGFβRI, p-TGFβRII^Y424^ and acetylated-α-tubulin (Ac-Tub) to identify the primary cilium. Faint p-TGFβRII^Y424^ staining was present throughout the cytoplasm but interestingly there was a higher concentration of p-TGFβRII^Y424^ along the entire length of the cilium (Fig. 2a), as previously reported for hESC^42^. This preferential ciliary localisation was verified by fluorescence intensity readings along the cilium demonstrating a 5.6-fold increase of intensity (p < 0.001, *n* = 9 ciliated cells) along the cilium when compared to the cytoplasm (Fig. 2b). A similar trend was also found with TGFβRI, with faint staining throughout the cytoplasm and strong concentration of TGFβRI along the primary cilium (Fig. 2c). The fluorescence intensity reading was 3.3-fold higher (p < 0.001, *n* = 7 ciliated cells) within the cilium when compared to the cytoplasm (Fig. 2d). Faint TGFβRI staining was also found within the nucleus. We further quantified the number of ciliated cells, which display this preferential spatial organisation and found that TGFβRI and p-TGFβRII^Y424^ co-localize with the cilium in 36.8 ± 26.5% and 76.3 ± 13.9% of ciliated cells (*N* ≥ 3, *n* ≥ 150 ciliated cells per group), respectively. These data demonstrate that TGFβ receptors are preferentially localised to the primary cilium in hMSCs, indicating that this organelle may be associated with regulation of TGFβ1 signalling and recruitment of hMSCs.

### TGFβ1-induced recruitment of hMSCs is mediated by the primary cilium

**Figure 3:**
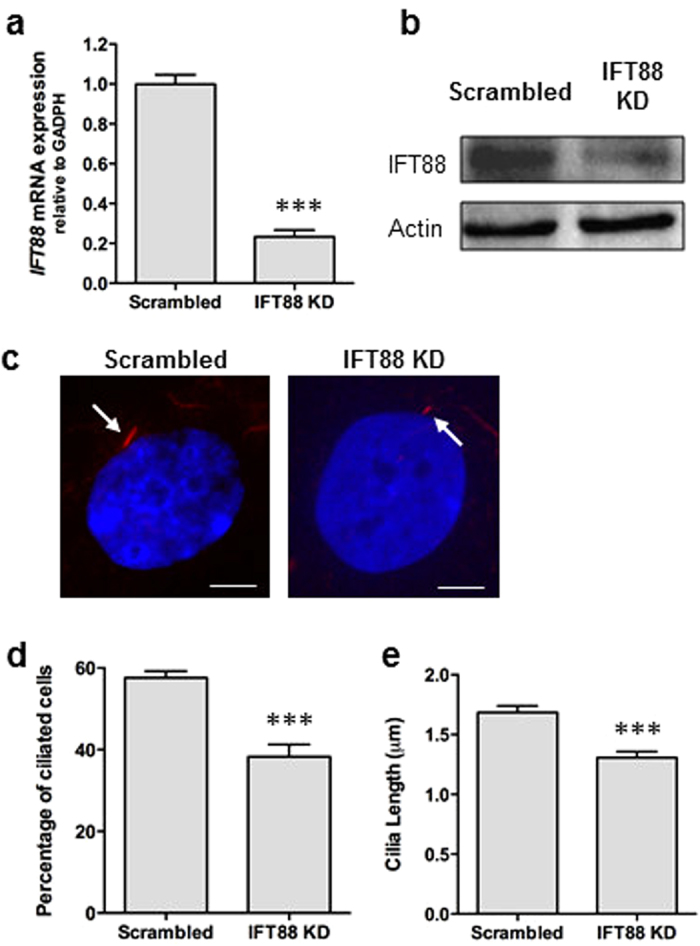
Knockdown of IFT88 in hMSCs impairs cilia formation/maintenance. (**a,b**) Efficiency of IFT88 siRNA transfection on *IFT88* mRNA expression analysed using qPCR (**a**) and Western blot (**b**). Expanded Western blot can be seen in Supplemental Figure S3. (**c**) Immunocytochemistry analysis of acetylated-α-tubulin (red) and DAPI (blue) showing shortened primary cilia (identified by arrows) after IFT88 KD. Scale bar 5 μm. (**d,e**) Analysis of the percentage of ciliated cells (**d**) and cilia length (**e**) after IFT88 KD. Statistical analysis using Student’s *t*-test. ***p < 0.001.

To determine whether the primary cilium is required for TGFβ1–induced recruitment of hMSCs, cells were subjected to siRNA-mediated knock-down (KD) of IFT88, which is required for anterograde IFT and thereby formation of the primary cilium^45^,^46^,^47^. Seventy-two hours following transfection, mRNA levels of *IFT88* were significantly reduced by 80% (p < 0.001, *N* = 5) when compared to control cells transfected with scrambled siRNA (Fig. 3a). These results were confirmed at the protein level with western blot analysis (Fig. 3b). To further confirm a disruption of IFT and ciliogenesis, primary cilium incidence and length was quantified by IFM analysis (Fig. 3c). Knock-down of IFT88 resulted in a significant decrease in number of ciliated hMSCs (p < 0.001, *N* = 14, *n* ≥ 260 cells per group) and, of the cells that still possessed a cilium, the cilium length was stunted in comparison to scrambled controls (p < 0.001, *N* = 6, *n* ≥ 60 ciliated cells per group), indicating a defect in IFT (Fig. 3d,e).

As high concentrations of TGFβ1 has been previously shown to alter cilia length by modulating IFT88 expression^48^ and cell morphology by modulating actin organisation, we analysed cilia incidence, cilia length, and the actin cytoskeleton in hMSCs following 30 min and 2 hrs treatment of TGFβ1 at 0.1 pg/ml concentration to mimic that at which we observed hMSC recruitment. 0.1 pg/ml TGFβ1 treatment did not influence cilia incidence, cilia length or actin organisation in hMSCs (Figure S1a–c). Furthermore, as IFT88 has also been shown to influence actin organization in chondrocytes which may influence cell migration^49^, the actin organisation was analysed following IFT88 KD and compared to scrambled control. There was no observable difference in hMSC actin organisation following IFT88 KD (Figure S1d).

**Figure 4:**
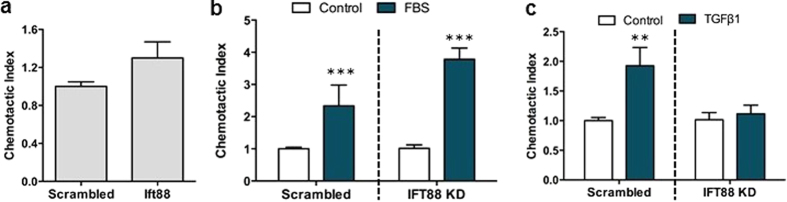
TGFβ1-induced hMSC chemotaxis requires the primary cilium. (**a**) Migration in the absence of chemotactic gradient of scrambled and IFT88 siRNA transfected cells. (**b**) Migration capacity of transfected cells towards 10% FBS presented as the chemotactic index expressed as mean ± SEM. (**c**) Chemotaxis of scrambled and IFT88 siRNA transfected hMSC towards 0.1 pg/ml TGFβ1. (**b,c**) Both scrambled and IFT88 KD are normalised to their respective no treatment control. Statistical analysis using Student’s *t*-test. **p < 0.01, ***p < 0.001.

Upon verification of a disruption of ciliogenesis, we next examined the migration capacity in IFT88-depleted cells. IFT88 KD did not result in any alteration in hMSC migration in the absence of a chemotactic gradient (i.e. identical media formulation in the upper and lower chamber) over the 18 hours of the experiment (Fig. 4a). Furthermore, in response to a gradient of 10% serum, scrambled siRNA-treated hMSCs responded with a 2.3-fold increase in migration (p < 0.001, *N* = 6) compared to serum free controls, which is in agreement with the positive control in Fig. 1a, demonstrating that the siRNA treatment alone does not influence hMSC migration. Furthermore, IFT88-depleted hMSCs also responded with a 3.8-fold increase in migration in response to 10% FBS (p < 0.001, *N* = 6), indicating that IFT88 is not required for serum-induced hMSC directional migration, as previously described for other cell types with defective ciliogenesis^26^. To determine whether primary cilia are required for TGFβ1–induced recruitment of hMSCs, we investigated the migration capacity of both scrambled and IFT88 siRNA-treated hMSCs in response to a gradient of 0.1 pg/ml TGFβ1. Scrambled siRNA hMSCs demonstrated a significant 1.9-fold increase in migration in response to TGFβ1 (p < 0.01, *N* = 18) which is in agreement with Fig. 1a. In contrast, TGFβ1-induced migration was blocked in IFT88-depleted hMSCs (Fig. 4c), demonstrating that primary cilia are required for the specific recruitment of hMSC induced by TGFβ1.

### Canonical TGFβ signalling components are localised and concentrated at the primary cilium in hMSCs

**Figure 5:**
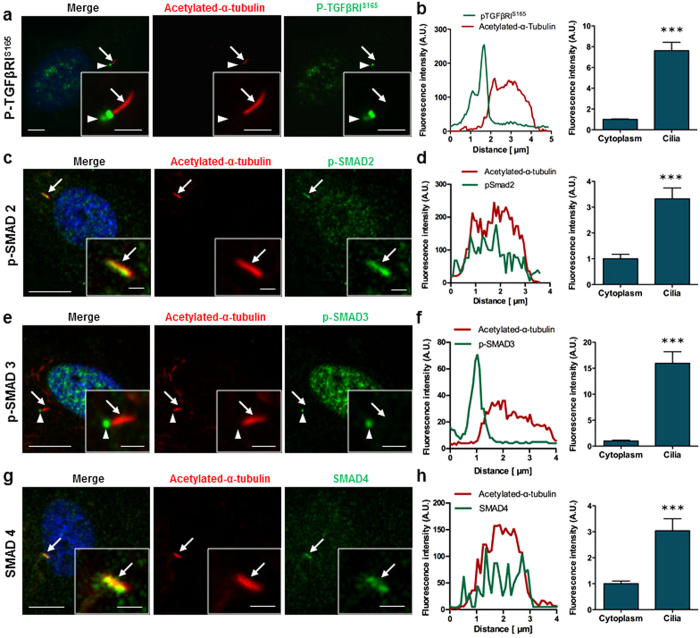
Canonical TGFβ signalling components localise to the primary cilium in hMSC. Immunofluorescence microscopy (IFM) of p-TGFβRI^S165^ (**a,b**) p-SMAD2 (**c,d**) p-SMAD3 (**e,f**) and SMAD4 (**g,h**). Primary cilia (identified by arrows) were stained with anti-acetylated-α-tubulin (red), nuclei with DAPI (blue) and p-TGFβRI^S165^ and all SMAD proteins are shown in green. Ciliary base is identified by arrowhead. Scale bars 5 μm and 2 μm (inserts). (**b,d,f,h**) Fluorescence intensity profile across the primary cilium and average fluorescence intensity analysis in the cilia compared to the cytoplasm. Statistical analysis using Student’s *t*-test. ***p < 0.001.

Since canonical TGFβ signalling operates through the phosphorylation of TGFβRI and SMAD transcription factors, we next examined whether TGFβ signalling components localize to the primary cilium, including phosphorylated forms of TGFβRI (p-TGFβRI^S165^)^50^, and receptor- SMADs 2 and 3 (p-SMAD2 and p-SMAD3) in addition to the common-mediator SMAD4, which oligomerizes with p-SMAD2/3 for their translocation to the nucleus and targeted gene expression in TGFβ signalling^37^. Similarly to the non-phosphorylated form of TGFβRI, faint p-TGFβRI^S165^ staining was present throughout the cytoplasm. However, unlike TGFβRI which co-localised along the length of the cilium, p-TGFβRI^S165^ accumulated at the ciliary base in 94.28 ± 6.3% of ciliated cells (Fig. 5a) (N = 3, *n* = 118 cells). Furthermore, the fluorescence intensity of p-TGFβRI^S165^ at the ciliary base is 3-fold higher than that within the cytoplasm, suggesting a higher concentration at the ciliary region (Fig. 5b) (N = 3, *n* = 10 cells). Phospho-SMAD2 was present throughout the cytoplasm with increased staining within the nucleus suggesting that there is a basal level of TGFβ signalling within hMSCs (Fig. 5c). Similar to that seen with TGFβRs, p-SMAD2 was found to co-localise with the primary cilium in 31.7 ± 40% of ciliated cells (*N* = 3, *n* = 140 cells). The fluorescence intensity of p-SMAD2 within the cilium was 3.3-fold higher than that of the cytoplasm (p < 0.001, *n* = 7 ciliated cells), (Fig. 5d). Furthermore, p-SMAD3 was also found throughout the cytoplasm but exhibited a more concentrated nuclear localisation. Interestingly, p-SMAD3 also co-localised to the primary cilium in 81 ± 9.6% of ciliated cells (Fig. 5e) (N = 11, *n* = 479 cells), but with intense concentrations at the ciliary base rather than along the cilium, demonstrating a 16-fold increase (p < 0.001, *n* = 7 ciliated cells) in relative levels compared to the cytoplasm (Fig. 5f), mirroring that which was observed with p-TGFβRI^S165^. Similar to p-SMAD2, SMAD4 was present throughout the cytoplasm and nucleus but also co-localised along the entire length of the cilium (Fig. 5g,h) with a 3-fold increase in intensity compared to the cytoplasm (p < 0.001, *n* = 9 ciliated cells) in 50.6 ± 32.7% of ciliated cells (*N* = 3, *n* = 147 cells). These data demonstrate that the phosphorylated form of TGFβRI and downstream effector SMAD proteins are preferentially localised to the primary cilium of hMSCs, with distinct spatial organisation within or at the base of the organelle.

### Disruption of the primary cilium inhibits TGFβ1-induced activation of SMAD3

**Figure 6:**
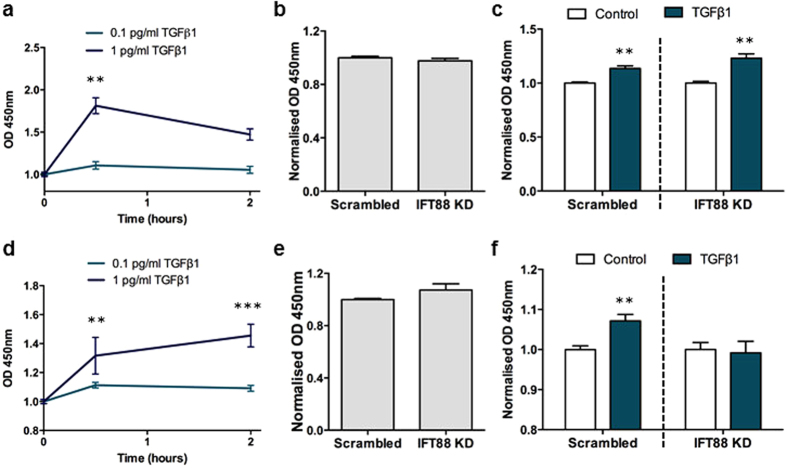
The primary cilium mediates SMAD3 signalling in hMSCs. (**a,c**) Analysis of the relative level of p-SMAD2/3 by ELISA. Quantification of the relative amount of p-SMAD2/3 with 0.1 pg/ml and 1 pg/ml TGFβ1 in control cells (**a**) and in cells transfected with Scrambled and IFT88 siRNA before (**b**) and after treatment with 1 pg/ml TGFβ1 for 30 minutes (**c**). Both scrambled and IFT88 KD are normalised to their respective no TGFβ1 treatment control. (**d–f**) Analysis of the relative quantity of p-SMAD3 by ELISA. Quantification of the relative amount of p-SMAD3 with 0.1 pg/ml and 1 pg/ml TGFβ1 in control cells (**d**) and in cells transfected with Scrambled and IFT88 siRNA without (**e**) and with treatment with 1 pg/ml TGFβ1 (**f**). Both scrambled and IFT88 KD are normalised to their respective no TGFβ1 treatment control. Statistical analysis using one-way Anova with Dunnett’s multiple comparison post-test (**a,d**), Student’s *t*-test (**b,c,e,f**). * p < 0.05, **p < 0.01, ***p < 0.001.

Previous studies showed that SMAD3 is primarily responsible for TGFβ1–induced migration of bone MSCs^29^. To investigate the role of the cilium in TGFβ-mediated SMAD signalling in hMSCs, we initially stimulated cells for 30 minutes and 2 hours with 0.1 pg/ml TGFβ1 followed by ELISA for quantification of phosphorylation levels of SMAD2/3 and specifically SMAD3 alone. Since no detectable change in phosphorylation of either SMAD2/3 or specifically SMAD3 was found at this low concentration of the ligand (Fig. 6a,d), we stimulated cells with 1 pg/ml TGFβ1, which also increases hMSC migration (Fig. 1a). In these experiments we observed a significant increase in phosphorylation of both SMAD2/3 and SMAD3 (p < 0.01, *N* = 3–5 for p-SMAD2/3, and p < 0.01, *N* = 8–10 for p-SMAD3) following 30 minutes stimulation (Fig. 6a,d). To determine whether the primary cilium is required for increased phosphorylation of these receptor SMADs, ciliogenesis was transiently inhibited by siRNA-mediated KD of IFT88 (Fig. 3). KD of IFT88 did not affect basal levels of p-SMAD2/3 in hMSCs when compared to scrambled controls (Fig. 6b), and upon stimulation with 1 pg/ml TGFβ1 for 30 minutes, both IFT88-depleted cells and scrambled controls responded with a significant increase in the phosphorylation of SMAD2/3 (p < 0.001, *N* = 4) (Fig. 6c). We next sought to determine whether the primary cilium is required for increased phosphorylation of receptor SMAD3 specifically. IFT88 KD did not affect basal levels of p-SMAD3 in hMSCs when compared to scrambled controls (Fig. 6e). However, upon stimulation with 1 pg/ml TGFβ1 for 30 minutes, IFT88 KD completely abolished the increase in TGFβ1-mediated phosphorylation of SMAD3 (Fig. 6f). In line with previous findings that TGFβ1–induced migration of MSCs is specifically mediated through the phosphorylation of SMAD3^29^, our results support the conclusion that the primary cilium is required for TGFβ1–induced migration of hMSCs through the activation of SMAD3 rather than SMAD2.

### TGFβ1-induced canonical signalling takes place at the ciliary base

**Figure 7:**
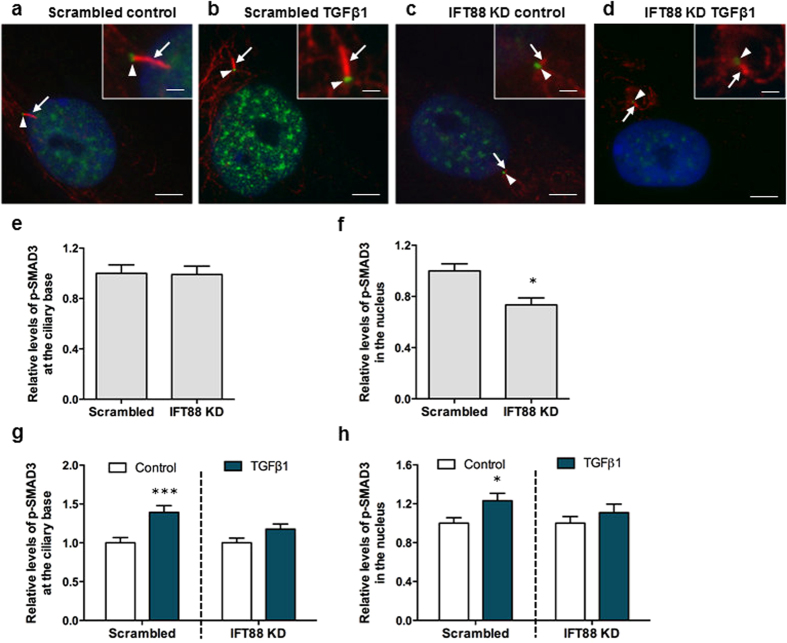
SMAD3 signalling takes place at the primary cilium and is mediated by IFT. (**a–d**) Representative immunofluorescence images of p-SMAD3 in Scrambled (**a,b**) and IFT88 siRNA transfected cells (**c,d**) before and after treatment with 1 pg/ml TGFβ1 for 30 minutes. Primary cilia were stained with anti-acetylated-α-tubulin (red, arrows), nuclei were stained with DAPI (blue) and p-SMAD3 protein is shown in green at the ciliary base region (arrowhead). Scale bars represent 5 μm and 1 μm (insert). (**e–f**) Analysis of the relative quantity of proteins at the (**e**) ciliary base region and (**f**) within the nucleus assessed by fluorescence intensities measurements of Scrambled and IFT88 transfected cells before treatment. (**g–h**) Analysis of the relative quantity of proteins at the (**g**) ciliary base region and (**h**) within the nucleus assessed by fluorescence intensities measurements of Scrambled and IFT88 transfected cells following treatment with 1 pg/ml TGFβ1 for 30 minutes. Both scrambled and IFT88 KD are normalised to their respective no TGFβ1 treatment control. Statistical analysis student’s *t*-test. *p < 0.05, ***p < 0.001.

To further establish the association of TGFβ signalling and the primary cilium in hMSCs, we finally sought to determine whether increased phosphorylation of TGFβRI and SMAD3 takes place at the base of the primary cilium and whether this is disrupted in cells subjected to IFT88 KD. hMSCs were treated with siRNA targeting IFT88 to impair ciliogenesis and scrambled siRNA as controls (Fig. 3) and p-TGFβRI^S165^ levels at the ciliary base (Fig. S2a–d) and p-SMAD3 levels at the ciliary base and within the nucleus (Fig. 7a–d) were quantified by IFM analysis. Interestingly, deletion of IFT88 resulted in a small but significant increase in p-TGFβRI^S165^ levels at the ciliary base (Figure S2e) (p < 0.001, *N* = 2, *n* ≥ 32 cells per group). p-TGFβRI^S165^ staining within the nucleus was very faint and therefore was not analysed. However, removal of the primary cilium did not affect basal levels of p-SMAD3 levels at the ciliary base (Fig. 7e) but significantly reduced localization of p-SMAD3 in the nucleus (p < 0.05, *N* = 3, *n* ≥ 41 cells per group) (Fig. 7f). Furthermore, following 1 pg/ml TGFβ1 stimulation for 30 minutes, the level of TGFβRI and SMAD3 phosphorylation increased at the ciliary base and p-SMAD3 levels increased within the nucleus in hMSCs transfected with scrambled siRNA (p < 0.001, p < 0.001 and p < 0.05 respectively, *N* ≥ 2, *n* ≥ 32 cells per group) (Figures S2f, 7g,h), demonstrating that canonical TGFβ signalling takes place at the base of the primary cilium. In contrast, IFT88 KD abolished TGFβ1-mediated increase in phosphorylations of TGFβRI at the ciliary base (Figure S2f) and SMAD3 at both the ciliary base (Fig. 7g) as well as in the nucleus (Fig. 7h). These data therefore demonstrate that TGFβ-induced canonical signalling occurs at the ciliary base followed by translocation to the nucleus and that this signalling pathway relies on functional IFT and formation of the primary cilium.

## Discussion

The recruitment of mesenchymal stem cells is a crucial process in the development, maintenance or repair of nearly every tissue throughout the body. TGFβ1 is a potent chemokine known to be essential for the recruitment of MSCs in bone, coupling the remodelling cycle. Dysregulation of this process has been linked to a number of skeletal pathologies^28^,^34^,^35^, which makes TGFβ1 a choice candidate for the development of therapeutics to promote MSC recruitment and restore this process. In this study we demonstrate that TGFβ1 induces the recruitment of human MSCs at low concentrations and, for the first time, established that the primary cilium mediates this recruitment process. Furthermore, we demonstrate that the primary cilium facilitates this through regulation of TGFβ1-mediated activation of SMAD3 via distinct spatial localisation of TGFβ receptors and signalling components at the cilium. Our findings therefore demonstrate a novel role for the primary cilium in the regulation of TGFβ signalling and subsequent migration of human MSCs.

Low concentrations of TGFβ1 significantly enhance recruitment but inhibit the osteogenic lineage commitment of hMSCs. This chemotactic response is in agreement with previous findings, whereby osteoclast-mediated bone resorption releases a gradient of TGFβ1, which induces the recruitment of progenitor cells to the bone surface^29^. Interestingly, this low concentration also resulted in an inhibitory effect on the osteogenic lineage commitment of hMSCs. Previous studies have demonstrated that TGFβ1 acts to inhibit late stage osteoblastic differentiation^28^,^43^,^51^. However, our dose response study discovered that this inhibitory effect occurs only at very low and high concentrations demonstrating a tri-phasic effect on MSC osteogenesis. The inhibition of osteogenesis at high concentrations may relate to a switch in lineage commitment to chondrogenesis^52^. At low concentrations, despite an inhibition of osteogenesis, Osteopontin (OPN) gene expression was increased which complies with finding by Zou *et al.*^53^ demonstrating a role for OPN in MSC migration. This may therefore suggest that low concentrations of TGFβ1 may maintain stemness to facilitate migration from the stem cell niche to the bone surface. The potent, yet diverse effect of TGFβ1 concentration on the regulation of different aspects of stem cell behaviour will provide useful information to regenerative medicine strategies utilising this growth factor. Our findings therefore support a model whereby a TGFβ1 gradient prevents osteogenic lineage commitment and supports the recruitment of MSCs to the area of need demonstrating the important role of this growth factor in MSC physiology.

Human MSCs which have defective primary cilia fail to home in response to TGFβ1 but interestingly maintain the ability for directional migration, demonstrating a novel chemokine specific role for the primary cilium in stem cell migration. Interestingly, recent findings by Chen *et al.* demonstrated a role for the cilium in MSC physiology, whereby in mice with defective osteoprogenitor primary cilia, bone formation is diminished due to a decrease in active mineralising surface^25^, suggestive of a defect in osteoprogenitor recruitment. Therefore, reduced bone formation in this model may be due to a defect in TGFβ1-induced MSC migration. Furthermore, previous work by Malone *et al.*^15^ has demonstrated that increases in OPN expression following mechanical stimulation is dependent on the primary cilium in pre-osteoblasts. Therefore TGFβ1-induced recruitment of hMSCs may be mediated by the primary cilium through regulation of OPN expression. The primary cilium has been implicated in the migration of other cell types such as fibroblasts, endothelial cells and neurons^26^,^54^,^55^. This phenomenon has been best described in fibroblasts where the cilium mediates the normal chemosensory response to PDGF-AA in wound healing^26^. This cilia-mediated migration was dependent on the ciliary localisation of the PDGF-AA receptor^56^. Hence, our data adds to the growing body of evidence linking the primary cilium to cell migration and introduces a novel stem cell recruitment mechanism.

Human MSCs preferentially localise receptors and downstream signalling components of the TGFβ pathway to the primary cilium, demonstrating a distinct spatial organisation within the cell. In this study, we have shown that TGFβ receptors I and II are localised to the primary cilium of human MSCs suggesting a role for this organelle as a chemosensor for the TGFβ1 ligand. Extending from the cell surface into the extracellular milieu, this ‘antennae-like’ organelle would facilitate receptor-ligand binding, and due to the distinct sub-compartment of the cilium, would enable efficient receptor multimerisation and subsequent phosphorylation of the downstream effector SMADs. Interestingly, we also observed localisation of p-SMAD2 and SMAD4 within the cilium and p-TGFβRI^S165^ and p-SMAD3 at the ciliary base. The different localisation of p-SMAD2 and SMAD4 within the ciliary compartment and p-TGFβRI^S165^ and p-SMAD3 at the base of the cilium may reveal different roles and regulation of these downstream signalling effectors. Clement *et al.*^41^ and Rys *et al.*^40^ recently demonstrated discrete spatial organisation of TGFβ receptors surrounding primary cilia and integrins respectively suggesting that this spatial localisation of receptors is a key mechanism of TGFβ regulation. Therefore, the stem cell primary cilia are ideally positioned and equipped to act as both a chemosensor and signalling centre for the TGFβ pathway.

TGFβ1-induced canonical signalling is mediated by the primary cilium, whereby SMAD3 phosphorylation occurs at the ciliary base. We found that TGFβ1 treatment leads to an increase in p-SMAD3 levels in hMSCs that was dependent on the primary cilium. This finding perfectly mirrors the effect of IFT88 KD on TGFβ1-induced stem cell migration. As TGFβ1-induced migration has previously been shown to be predominately dependent on SMAD3^29^, our data indicates that the primary cilium mediates stem cell recruitment via regulation of SMAD3 signalling. Interestingly, IFT88 KD did not influence the phosphorylation of SMAD2/3 following TGFβ1 stimulation. As this assay has a greater affinity to p-SMAD2 and given the inhibition of p-SMAD3 signalling with IFT88 KD, this data indicates that the primary cilium is not required for SMAD2 dependent TGFβ signalling. SMAD2 and SMAD3 have been shown to have diverse roles in many tissues such as the kidney^57^ and in breast cancer metastasis to bone^58^. Furthermore, 0.1 pg/ml TGFβ1 stimulation did not elicit a detectable change in canonical TGFβ signalling despite inducing a migratory response. Although this may be due to the sensitivity of the assays utilised, TGFβ1-induced migration at this concentration may be mediated by non-canonical signalling such as p-ERK1/2^41^. Primary cilia are known to coordinate many signalling pathways where ligands bind to receptors in the ciliary membrane and initiate signal transduction within the ciliary compartment^10^,^41^,^55^,^56^,^59^. We found that activation of TGFβ signalling resulted in an accumulation of p-TGFβRI^S165^ at the ciliary base and p-SMAD3 at the ciliary base and within the nucleus and this accumulation was once again dependent on the primary cilium. Interestingly, deletion of IFT88 resulted in a slight increase in p-TGFβRI^S165^ at the ciliary base in the absence of TGFβ but did not influence p-SMAD3 levels, indicating that a basal level of TGFβ signalling can occur independent of IFT, most likely initiating at focal adhesions as demonstrated by Rys *et al.*^60^. Our findings are commensurate to a model of cilia-mediated TGFβ signalling introduced by Clement *et al.* whereby upon ligand binding, TGFβRII/I, located along the cilium are trafficked to the ciliary base and undergo clathrin-dependent endocytosis facilitating phosphorylation of downstream SMADs at the ciliary base, followed by translocation to the nucleus^41^. Although receptor trafficking and receptor endocytosis at the ciliary base remains to be confirmed in human MSCs, the data presented here demonstrates that IFT88 regulates TGFβ signalling, demonstrating a novel mechanism of TGFβ pathway regulation.

This study presents evidence that TGFβ1 signalling through the primary cilium is necessary for the sensing of a TGFβ1 chemotactic gradient in human bone mesenchymal stem cells demonstrating a novel mechanism of TGFβ signal regulation and recruitment in stem cells. Therefore, this study highlights the primary cilium as a potential target to enhance MSC recruitment and bone formation in orthopaedic disease but also for other pathologies involving TGFβ1-induced MSC recruitment. Given the diverse roles of TGFβ1 in many tissues throughout the body, these findings may have important implications for several other pathologies.

## Methods

### Cell culture

Human bone marrow mesenchymal stem cells (hMSCs) from 2 different donors were obtained from ATCC (ATCC, PCS-500-012), who obtained the original cells under their ethics agreement. hMSCs were maintained in growth medium consisting of DMEM low glucose medium, 10% Fetal Bovine Serum (FBS, South America origin, Labtech) and 1% Penicillin-Streptomycin (P/S). For all experiments, cells were used from passage 3 to 5.

### Cell migration

Chemotaxis towards TGFβ1 was analysed using a modified Boyden chamber assay (24 wells Millicell inserts, 0.8 μm, Millipore). 10,000 cells were seeded within the inserts in a 24-well plate in DMEM without serum. After 4 hours, the inserts were transferred to a new plate containing serum free growth medium supplemented with 0.01 pg/ml to 10 ng/ml TGFβ1 (recombinant human, R&D systems). Cell migration was allowed for 18 hours and cells were fixed with 10% formalin and stained with haematoxylin. After extensive rinsing, the inside of the inserts were cleaned using cotton buds and the membranes were cut and mounted with DPX mounting medium. Migrated cells were quantified by bright field microscopy in at least 8 different fields of view at 20x and normalised to the number of cells migrated without TGFβ1 supplementation (chemotactic index).

### Cell proliferation

Human MSCs were seeded in 6 well plates at 30,000 cells/well, maintained for 2 days in growth medium followed by treatment with media containing 0.5% FBS supplemented with 0.01 pg/ml to 20 ng/ml TGFβ1. After 3 days, cells were rinsed twice with cold PBS and lysed on ice for 20 minutes with 100 μl lysis buffer containing 0.2% v/v Triton X100, 10 mM Tris pH8 and 1 mM EDTA. Cells were detached using a cell scraper, vortexed thoroughly and stored at −80 °C. The cell suspensions were homogenised using with a 21G needle before measuring DNA concentration using Quant-iT™ PicoGreen^®^ dsDNA Assay Kit (Thermoscientific) following the manufacturer protocol.

### Cell differentiation

Human MSCs were seeded in 6 wells plates at 30,000 cells/well and maintained in growth medium until 70% confluency is reached. The medium was then changed to osteogenic media containing 0.5% serum, 3.25 nM Dexamethazone, 5 mM β-glycerophosphate and 25 μM Ascorbic Acid. These concentrations are the minimum required to induce osteogenic differentiation^61^. TGFβ1 was added to the medium at 0.1 pg/ml to 20 ng/ml and the medium was replaced every 3 days for 14 days. Quantitative real-time PCR were performed with an ABI 7900 instrument (Applied Biosystems) using the protocol and primers described in Supplementary methods. For Alizarin red staining, cells were fixed at day 14 with 10% formalin for 15 minutes, washed and stained with 2% Alizarin red for 20 minutes and rinsed. Calcium ion quantification was performed using acidic extraction of Alizarin red based on a modified protocol from Gregory *et al.*^62^. Briefly, 10% acetic acid was added to the wells and incubated with shaking for 30 mins, the cell layer was scraped, transferred to a microcentrifuge tube and incubated at 85 °C for 10 minutes. Samples were then cooled on ice and centrifuged at 20,000 g for 15 minutes. 500 μl of the supernatant was mixed with 200 μl 10% (v/v) ammonium hydroxide and absorbance was measured at 405 nm. Calcium ion concentration was determined using standards of Alizarin red given that one calcium ion binds to two molecules of Alizarin Red S^63^.

### Immunostaining and co-localisation analysis

The localization of TGFβ1 signalling components within MSCs was investigated through immunocytochemistry after 48 hours of serum starvation (DMEM, 0.5% FBS, 1%P/S). Cells were fixed and stained for acetylated α-tubulin staining to identify the primary cilium, TGFβRII, TGFβRI and the downstream effectors phosphorylated TGFβRI (p-TGFβRI^S165^), phosphorylated SMAD2 (p-SMAD2), phosphorylated SMAD3 (p-SMAD3) and SMAD4 (Suppliers, references and dilution indicated in Supplementary methods). Coverslips were mounted with Fluoroshield-DAPI. To investigate the localisation of these components upon activation of the pathway, cells were treated with TGFβ1 (recombinant human TGFβ1, R&D Systems) for 30 minutes or 2 hours. All cells were imaged using an Olympus IX83 epifluorescence microscope fitted with 100x objective and a Zeiss LSM710 META Confocal Laser Scanning microscope fitted with a 63x objective. Control samples without secondary antibody were performed to ensure that there is no bleed-through in the experimental conditions. Intensity profiles along the cilium have been performed using Zen or CellSens software by tracing a line across the length of the primary cilia and measuring intensity along this line. Average intensities in the ciliary region, nucleus and cytoplasm were measured in three independent experiments on at least 7 cilia per condition. Region of interest were selected manually using ImageJ freeware and the intensities per pixel averaged. Regarding p-SMAD3 intensities analysis in transfected cells after TGFβ1 treatment, the cilia base region were selected automatically with ImageJ threshold tool for a minimum intensity of three times the average intensity in the cytoplasm (15 a.u.). The nuclear region were also selected automatically using the DAPI staining and the cytoplasmic region were selected manually close to the cilia and nucleus. Phospho-SMAD3 staining intensities were then measured in the regions of interest and both cilia and nucleus intensities were normalised to the intensity in the cytoplasm by removing the average intensity in the cytoplasm from each pixel of the region of interest. Measurements were performed in three different samples for at least 40 ciliated cells per condition.

### Measuring levels of SMAD signalling

Quantitative analysis of p-SMAD2 and p-SMAD3 was performed using PathScan^®^ Phospho-SMAD2 (Ser465/467)/Phospho-SMAD3 (Ser423/425) Sandwich ELISA Kit and PathScan^®^ phospho-SMAD3 (Ser423/425) Sandwich ELISA Kit (Cell Signaling Technology). Cells were grown in 100 mm Petri dishes and lysed using the buffer provided (Cell Signaling Technology #9803) complemented with 1 mM PMSF. Cell lysates were homogenised by passing the lysate through a 25G needle at least 10 times and centrifuged at 14000 g for 15 minutes at 4 °C. Total protein concentration was determined by BCA (Pierce™ BCA Protein Assay Kit, Thermofisher) to ensure the same quantity of protein were loaded in each well (30 to 50 μg). Briefly, SMAD2/3 Mouse Antibodies Coated Microwells were incubated with the sample diluted ½ in the sample buffer provided overnight at 4 °C. After rinsing, the wells were incubated with p-SMAD2/3 or p-SMAD3 Rabbit Detection Antibody for 1 hour at 37 °C. The presence of p-SMAD2/3 and p-SMAD3 were then detected with a HRP-linked secondary antibody and TMB substrate.

### Primary cilia knockdown

The formation of functional primary cilia was inhibited by small-interfering RNA (siRNA)-mediated depletion of IFT88, an intraflagellar transport protein (IFT) required for functional ciliogenesis. hMSCs were transfected with 32 μM siRNA targeting IFT88 (HSS111979, Invitrogen) or with a scrambled siRNA (Stealth RNAi™ siRNA Negative Control, Medium GC, 12935300, Invitrogen) for 24 hours using Lipofectamine^®^ RNAiMAX 1/250 (Invitrogen). Transfected MSC were maintained in DMEM 0.5% FBS for a further 24 hours before the cells were trypsinised to seed for experiments. Transfection efficiency was verified 72 hours after transfection by quantitative real-time PCR (qPCR), Western Blot and Immunocytochemistry. qPCR for *IFT88* mRNA was performed as described above. Western blot was performed as described in Supplementary methods for three different samples from two different experiments. Immunofluorescence were performed as described above for Acetylated-α-tubulin. Percentage of ciliated cells were determined and cilia length analysed by epifluorescence microscopy using an Olympus IX83 fitted with 100x objective. Cilia length were measured using Cellsens software (Olympus).

### Statistics

All data are presented as average ± S.E.M. and analysed using GraphPad Prism 5. Statistically significant differences were indicated as *p < 0.05, **p < 0.01, ***p < 0.001. For the dose-response studies one way Anova analysis were performed with Dunnett’s Multiple Comparison Test. All other analysis were performed using two-tailed unpaired student’s *t*-test. Regarding the migration assay, ELISA and intensities measurements of Ift88 KD cells, we observed a high variability between independent experiments and given the low TGFβ1 concentrations used induce a small but consistent effect, it was necessary to normalise the data to the controls for every single experiment.

## Additional Information

**How to cite this article**: Labour, M.-N. *et al.* TGFβ1 – induced recruitment of human bone mesenchymal stem cells is mediated by the primary cilium in a SMAD3-dependent manner. *Sci. Rep.* **6**, 35542; doi: 10.1038/srep35542 (2016).

## Supplementary Material

Supplementary Information
