# Supplementary material for: TGFβ1 – induced recruitment of human bone mesenchymal stem cells is mediated by the primary cilium in a SMAD3-dependent manner
Source: Sci Rep. 2016 Oct 17;6:35542. doi: 10.1038/srep35542 (PMC5066273; doi:10.1038/srep35542)
Supplement: Supplementary Information [file srep35542-s1.pdf]

TGFβ1 – induced recruitment of human bone mesenchymal stem cells is mediated by the primary cilium in a SMAD3-dependent manner

Marie-Noëlle Labour[1,2,4], Mathieu Riffault[1,2,3], Søren T. Christensen[5], David A. Hoey[1,2,3,4*]

[1]Trinity Centre for Bioengineering, Trinity Biomedical Sciences Institute, Trinity College Dublin, Dublin, Ireland.

[2]Department of Mechanical and Manufacturing Engineering, School of Engineering, Trinity College Dublin, Dublin, Ireland.

[3]Advanced Materials and Bioengineering Research Centre, Trinity College Dublin & RCSI, Dublin 2, Ireland

[4]Department of Mechanical, Aeronautical and Biomedical Engineering, University of Limerick, Limerick, Ireland.

[5]Department of Biology, University of Copenhagen, Copenhagen, Denmark.

*Corresponding Author: Tel: +353-1-8961359, email: dahoey@tcd.ie

Supplementary figures:

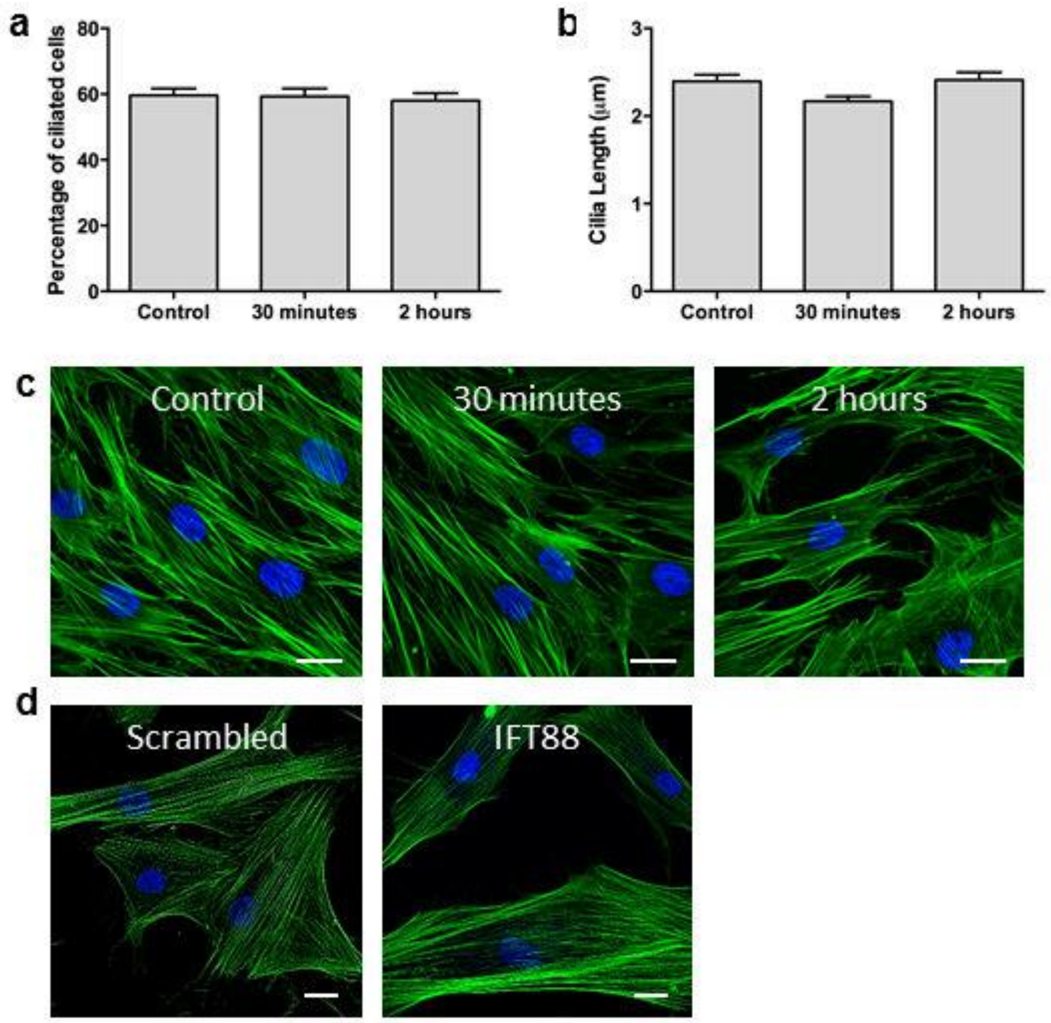

Figure S1: Effect of TGFβ1 and knockdown of IFT88 on hMSC primary cilium incidence, length and actin organisation. (a) Primary cilium incidence and (b) primary cilium length following treatment with 0.1pg/ml TGFβ1 for 30minutes and 2hrs. Representative immunofluorescence images of the actin cytoskeleton in hMSCs (c) following treatment with 0.1pg/ml TGFβ1 for 30 minutes and 2hrs and (d) following treatment with scrambled siRNA and siRNA targeting IFT88. Actin cytoskeleton was stained with Phalloidin (green) and nuclei were stained with DAPI (blue). Scale bar represent 20 μm.

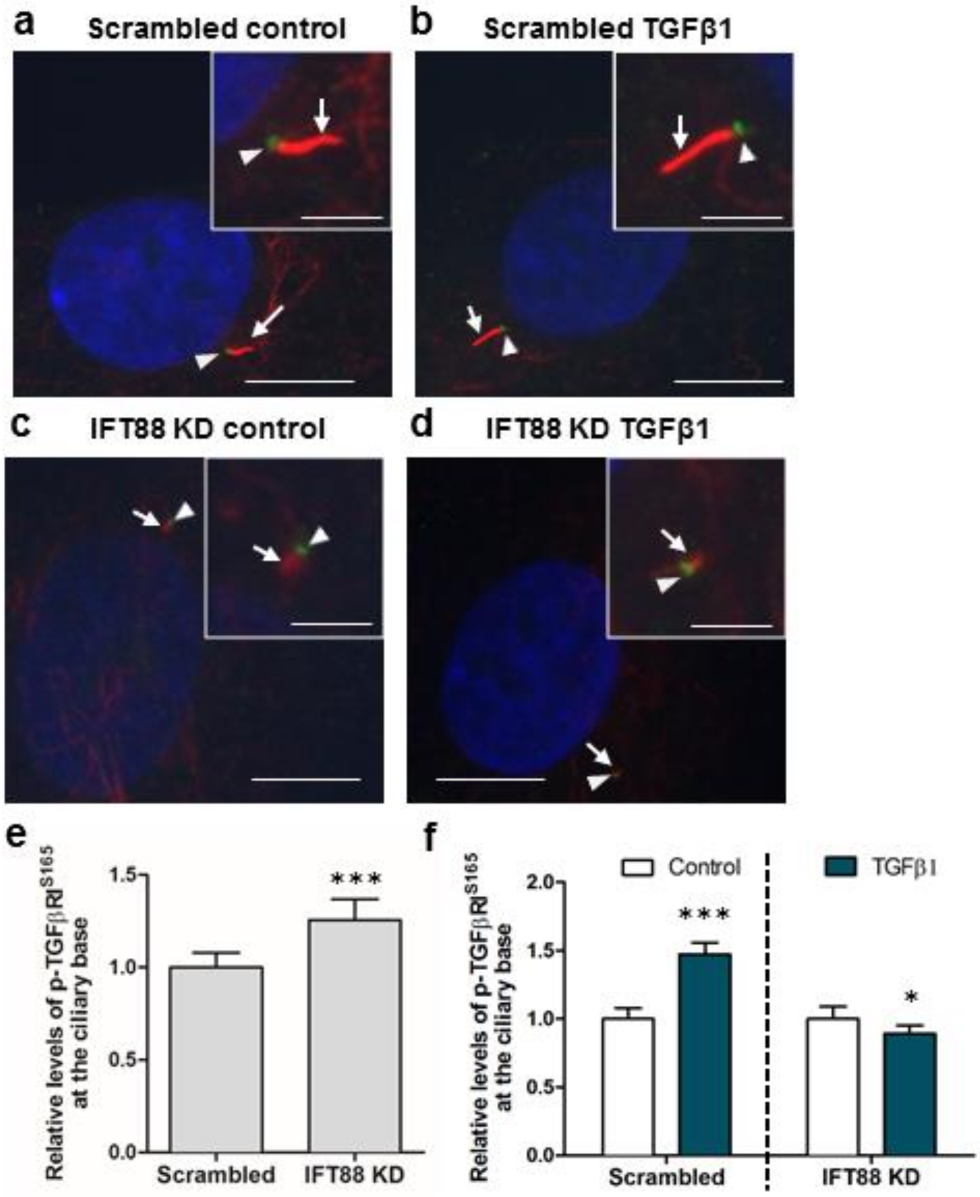

Figure S2: TGFβRI is phosphorylated at the primary cilium in an IFT-dependent manner. (a-d) Representative immunofluorescence images of p-TGFβRI^{S165} in Scrambled (a-b) and IFT88 siRNA transfected cells (c-d) before and after treatment with 1pg/ml TGFβ1 for 30 minutes. Primary cilia were stained with anti-acetylated-α-tubulin (red, arrows), nuclei were stained with DAPI (blue) and p-TGFβRI^{S165} is shown in green at the ciliary base region (arrowhead). Scale bars represent 5 µm and 1 µm (insert). (e-f) Analysis of the relative levels of p-TGFβRI^{S165} at the (e) ciliary base region assessed by fluorescence intensities measurements of Scrambled and IFT88 transfected cells before treatment. (f) Analysis of the relative quantity of proteins at the ciliary base region assessed by fluorescence

intensities measurements of Scrambled and IFT88 transfected cells following treatment with 1pg/ml TGFβ1 for 30 minutes. Both scrambled and IFT88 KD are normalised to their respective no TGFβ1 treatment control. Statistical analysis student's *t*-test. *p<0.05, *** p<0.001.

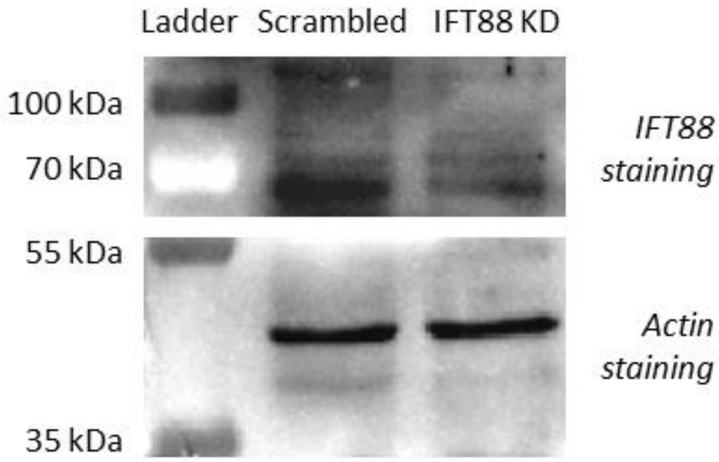

Figure S3: Expanded view of Western Blot probing for IFT88 as shown in Figure 3, demonstrating the knockdown of IFT88 at the protein level following transfection with siRNA targeting IFT88.

Supplementary methods:

List of antibodies:

Antibody	Supplier	Reference	Dilution
Acetylated α-tubulin	Abcam	ab24610	1/1500
p-TGFβRII (Y^{424})	Santa Cruz	sc17007R	1/500
TGFβRI	Santa Cruz	sc398	1/500
p-TGFβRI (S^{165})	Abcam	ab112095	1/300
p-SMAD2 (S^{465})	BioSS	BS0457R	1/300
p-SMAD3($S^{423/425}$)	Abcam	ab51451	1/500
SMAD4	Santa Cruz	sc7154	1/500

IFT88	Santa Cruz	Sc84318	1/200
Anti-rabbit IgG AlexaFluor 488	Life Technologies	A11008	1/500
Anti-mouse IgG AlexaFluor 594	Life Technologies	A21203	1/500
AlexaFluor488-Phalloidin	Life Technologies	A12379	1/40

RNA extraction, Reverse transcription and quantitative real-time PCR (qPCR): Cells were lysed with 1 ml TRIreagent (Sigma) and RNA was extracted following the manufacturer's instructions. Purity and concentration were determined by measuring absorbance at 260 and 280 nm using a nanodrop spectrophotometer. 1 µg RNA was reverse transcribed using High Capacity Reverse Transcription kit (4368813, Biosciences). The temperature cycles were as follows: 25°C for 10 min, 37°C for 120 min, 85°C for 5 min. Taqman qPCR were performed using Taqman universal PCR mastermix (Applied Biosystems 4304437) and 5ng of cDNA (5 µl final volume in 384-well plates) using an ABI 7900 instrument (Applied Biosystems). The primers used are listed below. qPCR was performed with an initial step of 10 min at 95°C followed by 40 cycles of 15 seconds at 95°C and 60 sec at 60°C.

List of primers (Invitrogen):

**Gene**	**Reference**
BMP2 650	Hs00154192_m1
RUNX2 860	Hs00231692_m1
Osteocalcin 632	Hs01587814_g1
Osteopontin 6696	Hs00959010_m1
Ift88	Hs00197926_m1
GAPDH	Hs02758991_g1

SDS-PAGE and Western blotting: Cells were grown and lysed as described for ELISA. The samples were concentrated by acetone precipitation and the total protein concentration were estimated using BCA assay following the manufacturer's instructions. Samples were diluted 1/5 in SDS reducing Laemmli sample buffer. Protein samples were then resolved by SDS-PAGE using the Mini-

PROTEAN® Tetra Cell Electrophoresis System (Bio-rad). 18 µg of proteins were loaded in 10% polyacrylamide gels along with the molecular weight ladder (Fermentas PageRuler™ Plus Prestained Protein Ladder, Thermoscientific Pierce) and the migration was run in Tris-Glycine-SDS running buffer. Proteins were then electrophoretically transferred to Amersham™ Protran Premium 0.2µm nitrocellulose membranes (Fisher Scientific) using the Bio-rad wet transfer system for 1.5 hours in Tris-Glycine Transfer buffer (25mM Tris base, 192 mM Glycine, 20% Methanol buffer). Membranes were blocked with 5% milk in TBST (Tris Buffer Saline – 0.1% Tween20) and stained overnight at 4°C for Actin (Cell Signaling Technology #4970, 1:1000) and IFT88 (Santa Cruz Technology sc-84318, 1:200) in TBST buffer containing 5% BSA. Blots were revealed using an anti-rabbit HRP antibody (Abcam ab97051, 1:40000 incubated 1 hour at room temperature) and ECL Select detection kit (Amersham RPN2235SK) according to the manufacturer's instruction. They were then imaged with the Gel Doc™ XR+ System (Biorad).
